# Effects of an overnight high-carbohydrate meal on muscle glycogen after rapid weight loss in male collegiate wrestlers

**DOI:** 10.1186/s13102-021-00325-w

**Published:** 2021-08-20

**Authors:** Emi Kondo, Keisuke Shiose, Takuya Osawa, Keiko Motonaga, Akiko Kamei, Kohei Nakajima, Hiroyuki Sagayama, Takahiro Wada, Shigeki Nishiguchi, Hideyuki Takahashi

**Affiliations:** 1 Sports Medical Center, Japan Institute of Sports Science, 3-15-1 Nishigaoka, Kita-ku, Tokyo 115-0056 Japan; 2 Department of Sports Research, Japan Institute of Sports Science, 3-15-1 Nishigaoka, Kita-ku, Tokyo 115-0056 Japan; 3 Faculty of Health and Sport Sciences, University of Tsukuba, 1-1-1 Tennodai, Tsukuba, Ibaraki 305-8577 Japan; 4 Faculty of Physical Education, Kokushikan University, 7-3-1 Nagayama, Tama-shi, Tokyo 206-8515 Japan; 5 Faculty of International Studies, Takushoku University, 815-1 Tatemachi, Hachioji-shi, Tokyo 193-0985 Japan

**Keywords:** Wrestling, Weight management, Recovery, Rapid weight loss, Rapid weight gain, Carbohydrate

## Abstract

**Background:**

Severe rapid weight loss (RWL) induces a decrease in muscle glycogen (mGly). Nevertheless, adequate carbohydrate intake after RWL has not been reported to optimize muscle glycogen following a weigh-in the evening until a wrestling tournament morning. The purpose of this study was to investigate the effect of an overnight high-carbohydrate recovery meal of 7.1 g kg^−1^ following RWL on mGly concentration.

**Methods:**

Ten male elite wrestlers lost 6% of their body mass within 53 h and then subsequently ate three meals, within 5 h, containing total of 7.1 g kg^−1^ of carbohydrates. mGly was measured by ^13^C-magnetic resonance spectroscopy before (BL) and after RWL (R0) at 2 h (R2), 4 h (R4), and 13 h (R13) after initiating the meal. Body composition, muscle cross-sectional area, and blood and urine samples were collected at BL, R0, and R13.

**Results:**

Body mass decreased by 4.6 ± 0.6 kg (*p* < 0.05) and did not recover to BL levels in R13 (− 1.7 ± 0.6 kg, *p* < 0.05). Likewise, mGly by 36.5% ± 10.0% (*p* < 0.05) and then did not reach BL levels by R13 (*p* < 0.05).

**Conclusion:**

A high-carbohydrate meal of 7.1 g kg^−1^ after 6% RWL was not sufficient to recover mGly during a 13 h recovery phase. Participating in high-intensity wrestling matches with an mGly concentration below normal levels is maybe undesirable.

## Background

As wrestling is a weight-categorised sport, wrestlers and coaches take advantage of body size and physical strength using rapid weight loss (RWL) strategies. Until 2017, a rule of United World Wrestling stated that an official weigh-in had to be conducted on the evening before each competition [1]. In 2018, this rule changed to requiring a weigh-in each morning [2]. In contrast, Judo changed the weigh-in timing from morning before each match to the evening before the match [3]. Other sports, such as professional boxing and mixed martial arts, still perform a weigh-in the day before the competition. Thus, there are some rules depend on the type of sports, and optimal weigh-in rules are still under argument.

Traditionally, many male wrestlers (38–69%), particularly in the lightweight and middleweight classes, attempt to “make weight” by losing > 5% of their body mass approximately one week before their competition and then ingest fluids and food to recover their physical condition as quickly as possible [4–6]. Common RWL methods include food and fluid restriction, fasting, eating less food during each meal, decreasing carbohydrate intake, increasing exercise, saunas and baths, and training with rubber suits [4, 5, 7]. These methods induce dehydration [8, 9], and decrease muscle glycogen (mGly) [10, 11]. As a result, aerobic and anaerobic performance decrease [12] and immune [13, 14] and cognitive functions are impaired [15]. However, wrestlers and coaches have considered that even if RWL reduces strength or endurance performance, these can be recovered through sufficient fluid and nutrition intake because the weigh-in was conducted in the evening before the day of each tournament. In addition, a recent review reported that an RWL of 5–8% body mass with a small impact on health and performance has remained acceptable practice [16]. Thus, if we accept RLW within a suitable range, the best nutrition strategies for recovery must be elucidated to achieve the best performance.

MGly is a major energy source of moderate- to high-intensity exercise [17]. Wrestling is an intermittent, combative sport requiring technical, tactical and psychological skill that demands absolute muscle strength and power on both the upper and lower body [18, 19]. Each match lasts approximately 6 min (two, 3 min rounds with a 30 s rest) including tackling, pushout, lifting, throwing, and blocking, which are high-intensity movements [20], and wrestlers must compete in up to five matches [21]. Therefore, the strategies of mGly recovery in the morning before the wrestling matches are important.

To the best of our knowledge, few studies, with only one manipulated carbohydrate intake after RWL, have investigated nutrient strategies to recover mGly overnight, which is generally the time between weigh-in and the first match. Only one study of Rankin et al. [22] reported the effects of carbohydrate intake from a high-carbohydrate meal (75% energy) compared with those of a moderate-carbohydrate meal (47% energy) for a 5 h recovery period on anaerobic performance. In the study, anaerobic performance tended to be higher after a high-carbohydrate meal than a moderate-carbohydrate meal, which was 99.1% and 91.5% of baseline after recovery, respectively, although mGly concentration was not obtained. As a nutrition guideline, the carbohydrate recommendation after or during exercise is described to be dependent on the exercise situation, such as intensity and duration [23]. However, the carbohydrates required to recover mGly between RWL and the match on the next morning remain to be elucidated.

The aim of this study was to examine the effect of a high-carbohydrate meal of 7.1 g kg^−1^ on the mGly concentration after RWL and an overnight recovery meal. To obtain the recovery process of mGly and short-term mGly synthesis, we measured these at 2 and 4 h after initiating the recovery meal. We hypothesized that a high-carbohydrate meal induces recovery of the mGly deficit after an overnight recovery phase.

## Methods

### Participants

Ten male collegiate wrestlers (age, 20.9 ± 0.5 year; height, 168.9 ± 4.3 cm; body mass, 73.2 ± 8.2 kg; % body fat, 11.2% ± 2.0%) were recruited from two teams in Japanese colleage after receiving information about this study from their team coaches. All subjects belonged to the East Japan Collegiate League and had competed in wrestling matches on an international (n = 3), national level (n = 5) and regional level (n = 2). Eligibility into the study required participants: (1) ≥ 18 years of age, (2) had experienced losing over 6% of their body mass before a major competition, (3) free of any metabolic, thyroid, or heart diseases. All participants submitted their written informed consent before the experiment began. This study was approved by the Institutional Review Board of the Japan Institute of Sports Sciences (036 in 2014).

### Experimental design

All participants were instructed to complete their daily life dietary and training records before the experiment. Participants visited our institute on the day before baseline (BL) measurements and ate their usual amount of dinner in a buffet restaurant by 21:00. They were given instructions to abstained from taking alcohol or stimulant beverages and to refrain from hard exercise for at least 12 h prior to BL measurement. They were allowed to drink mineral water after 23:00.

We obtained anthropometric measurements, body composition, and mGly concentration, and took blood and urine samples at 06:30 (BL). After the measurements, we instructed them to lose 6% of their body mass. The methods of weight loss is described in detail elsewhere [8]. At 53 h after BL measurements, they visited the laboratory for measurements after 6% RWL (R0). After the R0 measurements had been collected, the participants were provided with three prescribed meals (Table 1) to consume between 17:30 and 23:00, which was similar to previous study [24]. MGly concentrations were obtained 2 h (R2) and 4 h (R4) after initiating the prescribed meal. Measurements were taken again on the next morning at 06:30, 13 h after initiating the prescribed meal (R13).

**Table 1:** Time, menu, and nutritional intake of prescribed recovery meal

Period		R0–R2	R2–R4	R4–R13	Total
Time (hh:mm)		17:30–19:00	21:00–22:00	22:30–23:00	
Menu		Sports drink, Sports jully A bowl of rice topped with chicken and eggs, Hamburg, Miso soup with pork and vegetables, Baumkuchen, Water	Wheat noodle with seasoning soy sauce, A bowl of rice topped with chicken and eggs, 100% pure orange juice, Baumkuchen, Water	Baumkuchen, Water	
*Nutrient contents*					
Energy	(kcal)	1633 ± 210	1014 ± 164	231 ± 83	2879 ± 310
	(kcal kg^−1^)	22.3 ± 0.8	13.8 ± 1.5	3.2 ± 1.5	39.3 ± 1.0
Carbohydrate	(g)	290 ± 46	199 ± 23	33 ± 20	522 ± 47
	(g kg^−1^)	3.9 ± 0.2	2.7 ± 0.4	0.5 ± 0.3	7.1 ± 0.2
Protein	(g)	37 ± 5	23 ± 5	3 ± 1	64 ± 9
	(g kg^−1^)	0.5 ± 0.0	0.3 ± 0.0	0.0 ± 0.0	0.9 ± 0.0
Fat	(g)	36 ± 1	14 ± 9	10 ± 0	60 ± 10
Sodium	(mg)	2826 ± 216	1192 ± 429	58 ± 9	4076 ± 589
Food weight	(g)	2414 ± 431	1353 ± 334	917 ± 379	4685 ± 514
*Percentage of energy*					
Carbohydrate	(%)	70.8 ± 2.4	79.2 ± 7.3	54.5 ± 7.6	72.6 ± 1.4
Protein	(%)	9.2 ± 0.4	9.1 ± 1.1	5.6 ± 0.1	8.8 ± 0.3
Fat	(%)	20.0 ± 2.3	11.7 ± 6.7	39.8 ± 7.5	18.5 ± 1.1

Nutrient contents are described as mean ± SD (n = 10)

### Anthropometric data and body composition

All participants voided their bladders before measurements of height, body mass, and body composition were taken, and they each wore a bathing suit. Height and body mass were obtained barefoot to the nearest 0.1 cm with an automatic stadiometer and weight scale (A & D Co. Ltd, Tokyo, Japan) after voiding. Body composition was obtained using dual X-ray absorptiometry (DXA; QDR 4500, Discovery A [S/N 84498], fan-beam scanner, software version 12.7.3.2; Hologic, Waltham, MA, USA). Detail method of DXA is described in the previous study [8].

### Cross-sectional area measurements

The cross-sectional area (CSA) of the right femoral region was obtained with the participants in a supine position by 3-Tesla magnetic resonance imaging (MRI) device (Magnetom Skyra; Siemens, Erlangen, Germany) at each time point. First, localization images were obtained from the 3 anatomic planes (sagittal, coronal, transverse). The CSA of the muscles and subcutaneous fat in the femoral region was estimated at 50% position between the upper end of the greater trochanter and the knee joint gap (repetition time 500 ms, echo time 8.2 ms, matrix 256 × 256, field of view 240 mm, thickness 10 mm). Each CSA was computed by tracing each area using special image analysis software (ISIS, Hitachi Ltd., Japan) by one researcher.

### Muscle glycogen concentration

MGly concentration from right thigh was measured using carbon (^13^C)-magnetic resonance spectroscopy (MRS), as described elsewhere [25, 26]. To obtaine the ^13^C-glycogen signal, a 3-Tesla magnetic resonance system (Magnetom Verio; Siemens, Erlangen, Germany) with a ^13^C–^1^H double-tuned surface coil of 10 cm diameter (Takashima seisakusho, Japan) was used. The reproducibilityof mGly by ^13^C-MRS with repositioning and reshimming was coefficient of variation of 3.5% in our laboratory [25], and that of 6.6–9.5% in a previous study [27].

### Blood and urine sampling and analysis

Blood samples were collected from the antecubital vein into vacuum-sealed serum collection tubes and vacuum tubes containing dipotassium ethylene-diamine tetraacetic acid (EDTA) and sodium fluoride at three collection time points (BL, R0, and R13). The samples were centrifuged at 3000 rpm for 10 min at 4 °C, and whole blood, serum and plasma samples were stored at 2–4 °C, except plasma for arginine vasopressin, which was stored at − 80 °C until analysis. Urine samples for urinary specific gravity and osmotic pressure were obtained from each participant into 10 mL tubes and stored at 2–4 °C until analysis. All blood and urine analyses were outsourced to an independent laboratory (LSI Medience Corporation, Tokyo, Japan).

### Energy and macronutrient intake

A survey of all food and fluid intake was conducted before the baseline measurement (baseline) and during the 53 h RWL period (weight-loss period). We instructed participants to weigh all consumed food, supplements, and bevarages using a scale, and record these with photograph. Energy and macronutrient intake determined over the 3 days included 2 training days and 1 day off before the baseline measurement, and daily intake was calculated by adjusting for the training period as 6 days. Participants recorded all consumed food and fluid during the 53 h RWL period. The methods of calculation of energy and macronutrient intake is described in detail elsewhere [8].

### Statistical analysis

Sample size was calculated from the statistical power (1 − β) at 0.8, the α error at 0.05 and significant minimum effect size (f) at 0.6 based on the change in mGly concentration [11]. This power calculation determined that a minimum sample size of eight participants (for a repeated one-way ANOVA with five measurements) was required to detect a statistically significant difference in mGly concentration change using G*Power 3.1. Statistical analyses were performed using IBM SPSS statistics 24 for Windows (IBM, Chicago IL, USA) and expressed as means ± standard deviation. Paired t-test was used to compare mean difference between baseline and weight-loss period. Comparisons of means were performed using linear mixed model, and multiple comparison test was performed using Bonferroni post-hoc test.

## Results

### Nutrient intake and body mass

**Fig. 1:**
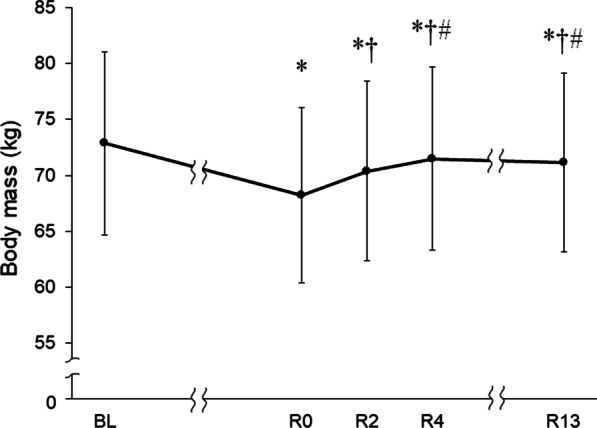
Change in body mass between rapid weight loss and recovery. Wrestlers underwent rapid weight loss (6% body mass reduction). Body mass was measured before (BL) and after rapid weight loss (R0) at 2 h (R2), 4 h (R4), and 13 h (R13) after initiating meal. Data are presented as means and SD (n = 10). *p < 0.05 versus BL; ^†^*p* < 0.05 versus R0; ^#^*p* < 0.05 versus R2

Daily energy intake decreased by 67.6% ± 11.2% during the weight-loss period compared with that of the baseline (Table 2). Body mass significantly decreased by 4.6 ± 0.6 kg (6.4% ± 0.7%) at R0 compared with BL (*p* < 0.05; 95% confidence interval, CI 4.2–5.2) (Fig. 1). Body mass then increased by 2.2 ± 0.5 kg after the first recovery meal (R0–R2) and by 3.3 ± 0.5 kg after the second (R2–R4), but body mass at R13 was 1.7 ± 0.6 kg (2.3% ± 0.8%) lower than BL.

**Table 2:** Daily nutritional intake

Component		Baseline	Weight-loss period
Energy	(kcal)	3426 ± 774	1123 ± 492
Carbohydrate	(g)	491 ± 138	156 ± 68
	(g kg^−1^)	6.7 ± 1.8	2.1 ± 0.9
Protein	(g)	122 ± 28	42 ± 20
	(g kg^−1^)	1.7 ± 0.3	0.6 ± 0.2
Fat	(g)	108 ± 22	37 ± 20
Sodium	(mg)	5181 ± 1307	1928 ± 632
Food weight	(g)	3673 ± 1512	1501 ± 654
*Percentage of energy*			
Protein	(%)	14.3 ± 0.8	15.1 ± 2.9
Fat	(%)	29.2 ± 4.7	29.3 ± 8.5
Carbohydrate	(%)	56.5 ± 4.9	55.6 ± 10.6

Values are expressed as mean ± SD (n = 10)

### Body composition and cross-sectional area of the right thigh

A mean fat mass reduction of 0.4 ± 0.3 kg (*p* < 0.05; 95% CI 0.1–0.8) and fat-free soft tissue reduction of 4.2 ± 0.3 kg (*p* < 0.05; 95% CI 3.8–4.6) were observed after RWL (R0) (Table 3). At R13, fat mass did not change from R0; however, the fat-free soft tissue recovered by 3.0 ± 0.4 kg (*p* < 0.05; 95% CI 2.6–3.4). Muscle CSA significantly decreased by 5.9% ± 1.3% (*p* < 0.05; 95% CI 9.4–12.2) from BL to R0 and increased by 3.8% ± 2.0% from R0 to R13 (*p* < 0.05, 95% CI 3.5–9.8), which was still lower than that of the BL (*p* < 0.05; 95% CI 1.8–6.4). There were no significant difference in the cross-sectional area of subcutaneous fat, bone and residual did not change at any point in time.

**Table 3:** Body composition and cross-sectional area of right thigh

	BL	R0	R13
*Body composition*			
Fat mass (kg)	8.4 ± 2.0	8.0 ± 1.8*	8.0 ± 1.9*
Fat-free soft tissue mass (kg)	63.2 ± 7.0	59.0 ± 6.7*	62.0 ± 6.6*^†^
Bone mineral mass (kg)	3.2 ± 0.3	3.2 ± 0.3	3.2 ± 0.3*
*Cross-sectional area*			
Muscle cross-sectional area (cm^2^)	180.8 ± 18.9	170.3 ± 19.3*	177.0 ± 18.0*^†^
Subcutaneous fat (cm^2^)	32.7 ± 10.6	31.2 ± 10.0	31.7 ± 10.0
Bone (cm^2^)	6.5 ± 0.7	6.4 ± 0.7	6.5 ± 0.7
Residual (cm^2^)	3.8 ± 0.7	3.9 ± 0.6	3.7 ± 0.5
Total cross-sectional area (cm^2^)	223.7 ± 21.9	211.8 ± 22.0*	218.8 ± 20.9*^†^
Circumference (cm)	48.4 ± 2.4	47.2 ± 2.6*	47.9 ± 2.3*^†^

Values are expressed as mean ± SD (n = 10). *BL* baseline. *p < 0.05 versus BL; ^†^*p* < 0.05 versus R0

Fat-free soft tissues mass excluded fat and bone mineral mass

### Muscle glycogen concentration

**Fig. 2:**
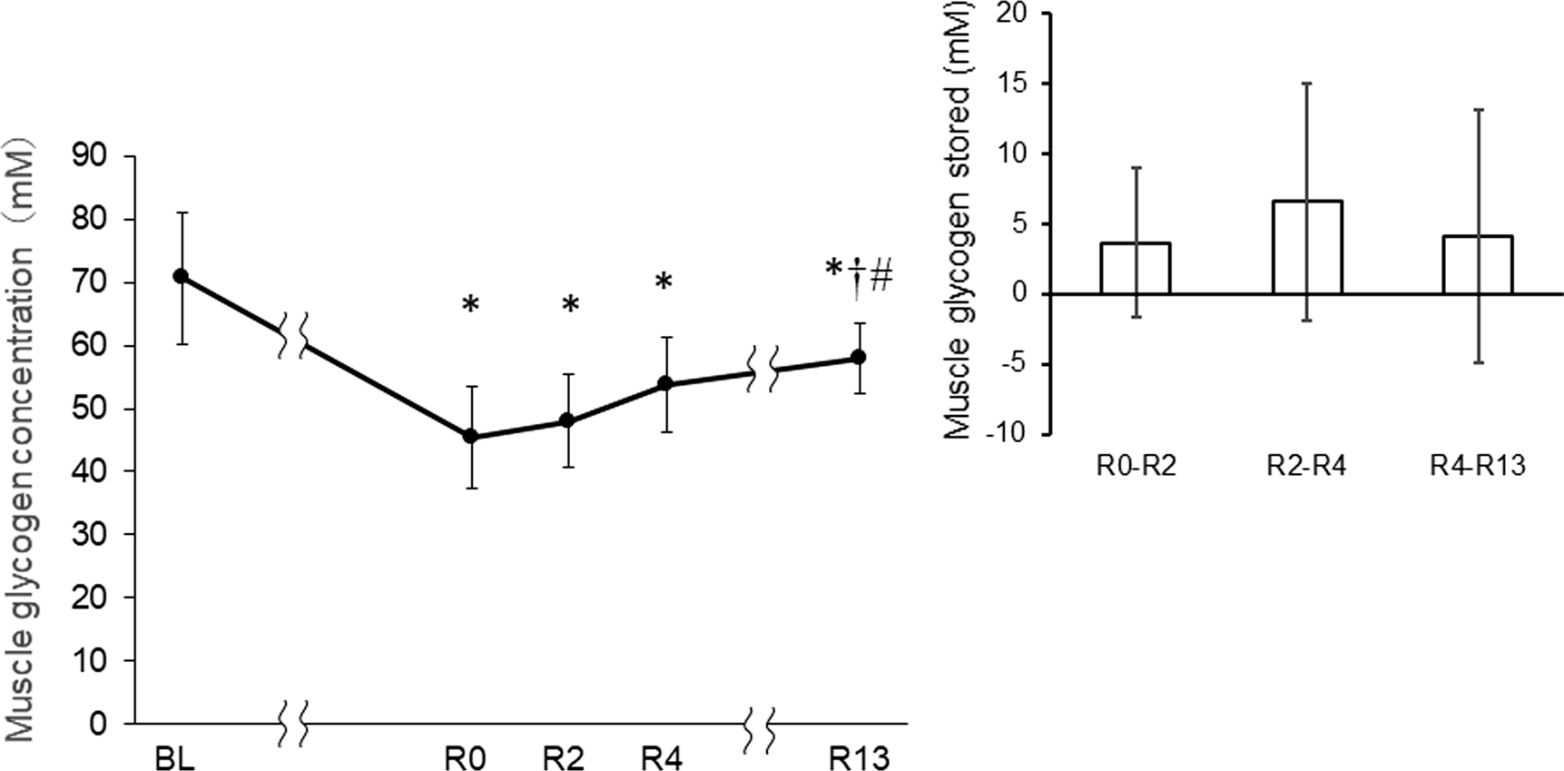
Change in muscle glycogen between rapid weight loss and recovery. Muscle glycogen was measured before (BL) and after rapid weight loss (R0) at 2 h (R2), 4 h (R4), and 13 h (R13) after initiating meal. Data are presented as means and SD (n = 10). BL, baseline; *p < 0.05 versus BL; ^†^*p* < 0.05 versus R0; ^#^*p* < 0.05 versus R2

MGly concentration decreased by 36.5% ± 10.0% at R0 compared with BL (*p* < 0.05; 95% CI 15.1–35.3) (Fig. 2), had not recovered by R2 (*p* < 0.05; 95% CI 14.5–30.7) or R4 (*p* < 0.05; 95% CI 2.8–30.7) and remained lower than BL (*p* < 0.05; 95% CI 1.5–23.8), even 13 h (R13) after initiating the prescribed meal containing 7.1 g kg^−1^ of carbohydrate. MGly store rate was 1.8 ± 2.7 mM h^−1^ from R0 to R2, 3.3 ± 4.2 mM h^−1^ between R2 and R4 and 0.5 mM h^−1^ between R4 and R13.

### Blood and urine content

Because one participant ate a dinner containing a higher fat content before pre-measurement compared with their usual meal, we analyzed the data from nine participants (Table 4). Hemoglobin, total protein, albumin, creatinine, uric acid, urea nitrogen, free fatty acid, total cholesterol, high-density lipoprotein (HDL)-cholesterol, low-density lipoprotein (LDL)-cholesterol, sodium, magnesium, and urinary specific gravity at R0 were greater than those at BL. By contrast, glucose, insulin, triglyceride, and testosterone were lower at R0 than at BL. Additionally, creatinine, uric acid, and triglyceride at R13 were statistically different compared with these measurements at BL.

**Table 4:** Change in biomarkers

Variable	BL	R0	R13	Reference ranges
*Blood*				
Hemoglobin (g dL^−1^)	15.0 ± 0.7	15.6 ± 0.9*	14.8 ± 0.7^†^	13.5–17.5
Hematocrit (%)	45.0 ± 1.9	46.5 ± 2.5	44.6 ± 1.7^†^	39.7–52.4
Total protein (g dL^−1^)	7.3 ± 0.3	8.2 ± 0.6*	7.2 ± 0.4^†^	6.7–8.3
Albumin (g dL^−1^)	4.6 ± 0.1	5.1 ± 0.3*	4.5 ± 0.1^†^	3.8–5.3
Creatinine (mg dL^−1^)	0.87 ± 0.11	1.06 ± 0.14	0.94 ± 0.11	0.61–1.04
Uric acid (mg dL^−1^)	5.5 ± 0.7	7.8 ± 1.1*	6.3 ± 1.0*^†^	3.8–7.0
Urea nitrogen (mg dL^−1^)	15.3 ± 4.5	22.5 ± 4.0	17.0 ± 2.7	8.0–20.0
Glucose (mg dL^−1^)	87 ± 5	81 ± 7*	87 ± 5^†^	70–109
Insulin (μU mL^−1^)	4.4 ± 1.9	2.3 ± 1.0	3.6 ± 1.3^†^	1.7–10.4
Triglyceride (mg dL^−1^)	118 ± 56	44 ± 12*	55 ± 11*	30–149
Free fatty acid (mEq L^−1^)	0.25 ± 0.05	0.73 ± 0.37*	0.35 ± 0.10^†^	0.10–0.90
Total cholesterol (mg dL^−1^)	179 ± 27	199 ± 25*	175 ± 20^†^	120–219
HDL-cholesterol (mg dL^−1^)	60 ± 8	71 ± 7*	62 ± 7^†^	40–85
LDL-cholesterol (mg dL^−1^)	97 ± 24	115 ± 23*	98 ± 17^†^	65–139
Sodium (mEq L^−1^)	140 ± 2	142 ± 1*	139 ± 2^†^	137–147
Potassium (mEq L^−1^)	4.7 ± 0.3	4.5 ± 0.2	4.7 ± 0.3	3.5–5.0
Chlorine (mEq L^−1^)	103 ± 1	104 ± 2	102 ± 2^†^	98–108
Magnesium (mEq L^−1^)	2.2 ± 0.1	2.5 ± 0.2*	2.2 ± 0.2^†^	1.9–2.5
Arginine vasopressin (pg mL^−1^)	9.1 ± 7.0	16.1 ± 8.2	10.4 ± 6.0	≤ 4.2
Testosterone (ng mL^−1^)	6.68 ± 1.45	4.75 ± 2.66*	6.27 ± 1.04^†^	1.92–8.84
*Urine*				
Urinary specific gravity	1.025 ± 0.010	1.037 ± 0.004*	1.026 ± 0.010^†^	1.006–1.030
Osmotic pressure (mOs mL^−1^)	886 ± 297	1166 ± 140*	809 ± 305^†^	50–1300

Values are expressed as mean ± SD (n = 9). *p < 0.05 versus BL; ^†^*p* < 0.05 versus R0

## Discussion

In the present study, we discovered that body mass and mGly concentrations do not return to pre-RWL levels after overnight recovery, even with the consumption of a high-carbohydrate meal (7.1 g kg^−1^ body mass) after 6% RWL. To our knowledge, this is the first study that obtained mGly changes during RWL and recovery using ^13^C-MRS. If we convert our mGly values to wet weight, assuming the density of fat-free mass is 1.1 kg L^−1^ [28], our basal values (69.0 ± 10.3 mmol kg^−1^ wet wt) agree with those of Houston et al. [10] (62 mmol kg^−1^ wet wt in the vastus lateralis). The mGly concentration decreased by 36.5% after 6% RWL for 53 h in the current study. The reduction in mGly concentration was also similar to that of Houston’s study (46% for a 96 h RWL of 8%). The mGly reduction rate calculated from the percentage of reduction and duration of RWL was 17% per day in the current study, 11% per day in Houston’s study. Thus, our results supported this previous study. Our participants’ intake of carbohydrate and protein were drastically less than that of BL values or dietary recommendations for weight loss [29]. Therefore, the amount of decrease in mGly was reasonable.

The fat-free soft tissue and muscle CSA decreased by 6.3% and 5.8% after RWL and recovered by 4.5% and 3.7% after taking the prescribed meal in the current study. These changes were similar to the study by Kukidome et al. [30], who reported that a lower muscle area decreased by 5.9% after 7.0% weight loss for 1-week and regained by 4.2% by the wrestling tournament. Our participants experienced dehydration such as fluid restriction and sweating with rubber suits during RWL, which affect total body water and the percentage of water in fat-free mass [8]. Indeed, several blood and urine indexes indicated dehydration (total protein, albumin, uric acid, sodium, magnesium and urinary specific gravity) at R0. Therefore, decrease in fat-free soft tissue and muscle CSA may be mainly associated with a decrease in hydration status in fat-free soft tissue. However, increase in the muscle CSA likely reflects rehydration in fat-free soft tissue and increase in intracellular water by glycogen accumulation. Most of blood and urine indexes of R13 returned to BL in the current study. In addition, previous studies suggested that mGly loading increases in muscle CSA of the vastus muscles and was probably due to an increase in intracellular water binding to glycogen granule [26, 31]. Furthermore, we found that total body water changed after RWL and recovery in the previous study [8], indicating that these body water changes might relate to mGly change.

RLW-induced reduction in mGly concentration did not again reach BL values after 13 h recovery in the current study. Most studies have examined short-term (0–6 h) or long-term (> 24 h) recovery strategies, and few studies examined a 12 h mGly recovery. Piehl et al. [32] reported that mGly recovered to 68.8% of at-rest values at 10 h after exhaustion (18% of mGly at rest), and it took 46 h to recover to rest levels. The calculated means of carbohydrate intake in their study were 216 g (5.1 g kg^−1^) for a 10 h recovery and 1116 g (15.9 g kg^−1^) for a 46 h recovery. Although the mGly restoration in current study (81.7%) was higher than the previous study [32], if athletes could consume more carbohydrates for longer periods, such as in professional boxing or mixed martial arts (e.g., 24 h), mGly recovery would likely have been enhanced. The mGly storage rate was 1.8 mM h^−1^ from R0 to R2, 3.3 mM h^−1^ from R2 to R4, and 0.5 mM h^−1^ from R4 to R13 (mean, 1.1 mM h^−1^ for a 13 h recovery). MGly accumulation calculated from Houton’s study [10] was 1.6 mmol kg wet wt^−1^ h^−1^ for a 3 h recovery, which is similar to the current study. However, these values are lower than the mGly accumulation range of 5–10 mmol kg wet wt^−1^ h^−1^ after extreme endurance exercise [33]. Therefore, it was considered that the recovery of mGly may be lower after RWL than after endurance exercise.

We considered several reasons for the low mGly storage in the current study. First, glucose transporter isoform (GLUT-4) relates closely to glucose uptake. During and after endurance exercises, muscle contraction increases insulin-independent glucose uptake to muscle by GLUT-4 translocation. However, in the present study, we expected translocated GLUT-4 to be less than that after exhaustive exercise because the participants did not eat the recovery meal immediately after exercising. GLUT-4 mediated glucose uptake is strongly affected by insulin, that effect is larger than insulin-independent glucose uptake [34]. Participants in the current study were wrestlers who were well trained and ingested large amounts of carbohydrates after RWL. Hence, we believe that the difference in insulin-independent glucose uptake after exercise is rarely relevant. Second, mGly reduction after RWL was lower (36.5%) than that of previous studies (67–91%) [32, 35]. Zachwieja et al. [36] reported that mGly and glycogen synthase activities were negatively correlated [36]. Indeed, in our study, mGly concentration at R0 was 45.4 mM, which was higher than that in previous post-exercise studies [35, 37]. Third, the amount of carbohydrate intake may affect mGly restoration. Although a recent review recommended 7–10 g kg^−1^ of carbohydrates during an overnight recovery period after RWL [16], 7.1 g kg^−1^ of carbohydrate may be insufficient to restore mGly. In the current study, serum insulin levels were decreased by RWL and recovered by consuming a high-carbohydrate meal. Furthermore, free fatty acid levels increased at R0 and returned at R13. These data suggest that RWL induced gluconeogenesis, and we expect that not only mGly but also liver glycogen was decreased. Therefore, more carbohydrates may be needed to fill glycogen in both the liver and muscle. Finally, we considered that the macronutrient balance affects mGly storage after RWL. Previously, Burke et al. [38] provided evidence that when the carbohydrate intake is < 1.2 g kg^−1^ h^−1^, the addition of protein enhances post-exercise mGly synthesis. By contrast, the fat content (60 g, 18.5% energy) in the current study may prevent carbohydrate absorption. Eating high glycemic index carbohydrate foods after prolonged exercise induces a greater recovery of mGly concentrations than low glycemic index carbohydrate foods [39]. Therefore, future studies should attempt to compare the meal-contained manipulated macronutrient.

This study has some limitations. First, as we did not measure physical performance, the relationship between high-carbohydrate intake after RWL and physical performance was equivocal. Second, we did not clarify the mechanism of mGly synthesis because interrupted blood samples and muscle samples could not be obtained during the recovery phase. Furthermore, since we only measured mGly in the right thigh, changes in mGly in other parts, such as arms or liver, were not clarified. Third, we did not have a control group, and we performed only one carbohydrate intervention. Therefore, further study is required to examine whether the amount of carbohydrate after RWL is associated with mGly recovery. Finally, we had estimated appropriate sample size by repeated one-way ANOVA before the experiment. Post-hoc test by the linear mixed model was justified enough power at 1.7 using Rstudio.

## Conclusions

We conclude that a high-carbohydrate meal of 7.1 g kg^−1^ body mass after 6% RWL was insufficient to recover the mGly during a 13 h recovery phase. Therefore, to avoid wrestling match with lower mGly level, optimal nutritional recovery strategies need to be further investigated.

## Abbreviations

ANOVA: Analysis of variance
CSA: Cross-sectional area
DXA: Dual X-ray absorptiometry
EDTA: Ethylene-diamine tetraacetic acid
GLUT: Glucose transporter
HDL: High-density lipoprotein
LDL: Low-density lipoprotein
mGly: Muscle glycogen
MRI: Magnetic resonance imaging
RWL: Rapid weight loss
